# The Effect of Aerobic Exercise on Cognitive Function in People with Alzheimer’s Disease: A Systematic Review and Meta-Analysis of Randomized Controlled Trials

**DOI:** 10.3390/ijerph192315700

**Published:** 2022-11-25

**Authors:** Shiyan Zhang, Kai Zhen, Qing Su, Yiyan Chen, Yuanyuan Lv, Laikang Yu

**Affiliations:** 1 Key Laboratory of Physical Fitness and Exercise, Ministry of Education, Beijing Sport University, Beijing 100084, China; 2 Department of Sports Performance, Beijing Sport University, Beijing 100084, China; 3 Ersha Sports Training Center of Guangdong Province, Guangzhou 510100, China; 4 China Institute of Sport and Health Science, Beijing Sport University, Beijing 100084, China

**Keywords:** aerobic exercise, Alzheimer’s disease, cognitive function, mini-mental state examination

## Abstract

A growing body of research has examined the effect of aerobic exercise on cognitive function in people with Alzheimer’s Disease (AD), but the findings of the available studies were conflicting. The aim of this study was to explore the effect of aerobic exercise on cognitive function in AD patients. Searches were performed in PubMed, Web of Science, and EBSCO databases from the inception of indexing until 12 November 2021. Cochrane risk assessment tool was used to evaluate the methodological quality of the included literature. From 1942 search records initially identified, 15 randomized controlled trials (RCTs) were considered eligible for systematic review and meta-analysis. Included studies involved 503 participants in 16 exercise groups (mean age: 69.2–84 years) and 406 participants (mean age: 68.9–84 years) in 15 control groups. There was a significant effect of aerobic exercise on increasing mini-mental state examination (MMSE) score in AD patients [weighted mean difference (WMD), 1.50 (95% CI, 0.55 to 2.45), *p* = 0.002]. Subgroup analyses showed that interventions conducted 30 min per session [WMD, 2.52 (95% CI, 0.84 to 4.20), *p* = 0.003], less than 150 min per week [WMD, 2.10 (95% CI, 0.84 to 3.37), *p* = 0.001], and up to three times per week [WMD, 1.68 (95% CI, 0.46 to 2.89), *p* = 0.007] increased MMSE score significantly. In addition, a worse basal cognitive status was associated with greater improvement in MMSE score. Our analysis indicated that aerobic exercise, especially conducted 30 min per session, less than 150 min per week, and up to three times per week, contributed to improving cognitive function in AD patients. Additionally, a worse basal cognitive status contributed to more significant improvements in cognitive function.

## 1. Introduction

Dementia is becoming a growing risk factor among the elderly population as the world’s population grows and the problem of population aging intensifies. As of 2019, there were approximately 57.4 million people worldwide with dementia, and it is estimated that there will be more than 152 million in 2050 [1]. This trend will continue to intensify as COVID-19 impacts the public health and healthcare system around the world [2]. Alzheimer’s disease (AD) is the most common form of dementia, accounting for 60%–80% of dementia patients [3,4].

AD is an insidious progressive neurodegenerative disease, typically characterized by memory impairment, cognitive impairment, and limitations in daily activities. Atypical symptoms are language, vision, and practical or executive problems that often begin at the onset of symptoms and cannot be effectively identified, leading to further disease progression [5]. This deterioration can lead to neuronal damage in the brain, severe memory and cognitive impairments that interfere with daily primary physical activity, and the loss of the ability to take care of oneself, leading to death [6]. It is worth noting that AD patients require caregivers for a long time, resulting in a huge medical burden and economic burden [7,8,9]. The total global cost of dementia is projected to reach $9.12 trillion in 2050 [10]. Risk factors for AD mainly include advanced age, family history, diabetes, middle-aged hypertension, smoking, alcohol consumption, apolipoprotein E (apoE) Ɛ4 allele, depression, physical inactivity, and low educational achievement [11,12]. Thankfully, some of these risk factors are modifiable.

Due to the numerous risk factors for AD and its complex pathological mechanism, current treatment focuses on improving patients’ symptoms and delaying AD progression. The most extensively studied treatment modalities for AD patients are pharmacotherapy and behavioral interventions [13,14]. Although many institutions have invested in pharmaceutical research to treat AD, the primary goal of pharmacotherapy is to improve the cognitive function and psychological status of the patient. Pharmacological treatment can only provide relief but cannot effectively control the deterioration of the disease and may be accompanied by side effects [15,16]. Therefore, non-pharmacological treatments (NPTs) have gained extensive attention in AD treatment. The main treatments for NPTs include lifestyle interventions, dietary modifications, exercise, and multimodal mixed interventions [14,17,18,19].

Studies have shown that exercise can improve the activities of daily living in older adults and delay cognitive decline in people with AD [20]. Smith et al. [21] have shown that aerobic exercise moderately improved neurocognitive function in healthy older adults, including improvements in attention and processing speed, executive function, and memory, which was consistent with Zheng et al. [22]. A study by Hindin et al. [23] showed that aerobic exercise was as effective as cognitive expansion practice in improving cognitive function in older adults. Guadagni et al. [24] conducted a 6-month aerobic exercise intervention with 206 healthy older adults, and the results showed that the aerobic exercise intervention significantly improved cognitive functions in verbal memory, graphic memory, and complex memory, and found that cognitive improvement may be related to cerebrovascular modulation brought about by exercise. In addition, a study by Ambrose et al. [25] showed that aerobic exercise three times a week for 6 months improved performance on the Alzheimer’s Disease Assessment Scale-Cognitive subscale (ADAS-cog) in adults with mild subcritical biochemical cognitive improvement, but cognitive function did not improve at 6-month follow-up. Nocera et al. [26] found that 6 months of high-intensity aerobic exercise could improve cognitive function in elderly patients with mild cognitive impairment, with significantly better benefits for women than men. Yang et al. [27] found that moderate-intensity aerobic exercise improved cognitive function in patients with mild AD. Moreover, Kemoun et al. [28] conducted a 15-week exercise intervention in 38 subjects; the results showed that aerobic exercise delayed the decline in cognitive function and improved exercise efficiency in patients with AD. Bernardo et al. [29] believed that endurance exercise could increase the antioxidant potential of the body, thereby reducing the neuropathological condition of AD patients. This evidence supports the idea that exercise can improve cognitive function in AD patients.

Although exercise is thought to improve cognitive function to some extent in AD patients, previous reviews have not focused on specific types of exercise, resulting in substantial heterogeneity between interventions. In addition, it seems that there are a lot of tools and scales for evaluating cognitive function in AD patients, but there are no globally accepted standards as to which ones are most important. Therefore, we conducted a comprehensive systematic review and meta-analysis of randomized controlled trials (RCTs) to explore the effect of aerobic exercise on cognitive function [as measured by mini-mental state examination (MMSE)] in patients with AD.

## 2. Methods

### 2.1. Design

Cochrane Selection Manual [30] and the Preferred Reporting Items for Systematic Reviews and Meta-Analysis (PRISMA, 2020) guidelines [31] were followed for conducting this systematic review and meta-analysis. The protocol was registered on PROSPERO (http://www.crd.york.ac.uk/PROSPERO, accessed on 22 June 2022), registration number: CRD42022340669.

### 2.2. Search Strategy

Original articles on aerobic exercise and cognitive function in AD patients were searched in three electronic databases: PubMed, Web of Science, and EBSCO, through November 12, 2021. The initial search contained the following terms: (a) exercise, aerobic exercise, endurance exercise, aerobic training, endurance training, cardio training, physical endurance, physical exertion; (b) cognition, cognitions, cognitive function, cognitive functions, function cognitive, functions cognitive; (c) Alzheimer disease, Alzheimer dementia, dementia Alzheimer, Alzheimer’s disease, dementia senile, senile dementia, dementia Alzheimer type, Alzheimer type dementia, dementia Alzheimer type, Alzheimer type senile dementia, primary senile degenerative dementia, dementia primary senile degenerative, Alzheimer sclerosis, sclerosis Alzheimer, Alzheimer dementias, Alzheimer syndrome, Alzheimer’s diseases, Alzheimer diseases, senile dementia Alzheimer type. In addition, the reference lists of all identified studies were manually searched to identify potentially eligible studies. Two authors (S.Z. and K.Z.) independently evaluated the retrieved articles using a standardized form. Discussions with the third author (L.Y.) resolved the disagreements.

### 2.3. Eligibility Criteria

Inclusion criteria were as follows: eligible studies (1) should be RCTs; (2) should include both an intervention and control group; (3) should use AD patients as subjects; and (4) should use MMSE as the primary outcome measure.

Exclusion criteria were as follows: (1) non-English language publications; (2) reviews and conference articles; (3) animal model publications; (4) the control group received other treatments.

### 2.4. Data Extraction

Two authors independently extracted the data from each study included first author’s last name, year of publication, sample size (*n*), intervention features (frequency, intensity, duration of intervention, session duration), participant characteristics (age and basal cognitive status), and treatment effects.

### 2.5. Methodological Quality Assessment

The quality of the included studies was evaluated using the Cochrane Collaboration tool according to the 7 domain biases as follows: (1) randomization sequence generation (selection bias), (2) allocation concealment (selection bias), (3) blinding of participants and personnel (performance bias), (4) blinding of outcome assessment (detection bias), (5) incomplete outcome data (attrition bias), (6) selective reporting (reporting bias), and (7) other bias [30]. Methodological quality assessment was performed independently by two authors. Discussions with the third author resolved the disagreements. The methodological quality of all the included studies was assessed by using a Physiotherapy Evidence Database (PEDro) Scale [32,33]. The PEDro Scale is an 11-item scale used to evaluate the quality of the RCTs of the physical therapy studies. The total Physiotherapy Evidence Database scores for RCTs were ranged from 0–10. Scores above 9, between 6 and 8, between 4 and 5, and below 4 are considered excellent, good, average, and poor quality, respectively [34].

### 2.6. Statistical Analysis

We calculated the changes in mean and standard deviation (SD) values of MMSE score. For studies reporting standard error (SE) or 95% confidence intervals (CIs), SD was calculated [22,35]. Data were pooled using random-effects models to obtain the weighted mean difference (WMD) and 95% CIs. If there was a high heterogeneity (*I*^2^ > 50%), subgroup analysis or sensitivity analysis was used to interpret the results [36,37]. In the subgroup analyses, we tried to use minutes of intervention per session (30 min per session and more than 30 min per session), minutes of intervention per week (less than 150 min per week and 150 min or more per week), frequency of intervention per week (up to three times per week and more than three times per week), and basal cognitive status of participants (mild and moderate) to explore the impact on cognitive function. The analysis result, funnel plot, and forest chart were generated using the software RevMan.5. In terms of overall impact, *p* < 0.05 was considered statistically significant.

## 3. Results

### 3.1. Study Selection

**Figure 1:**
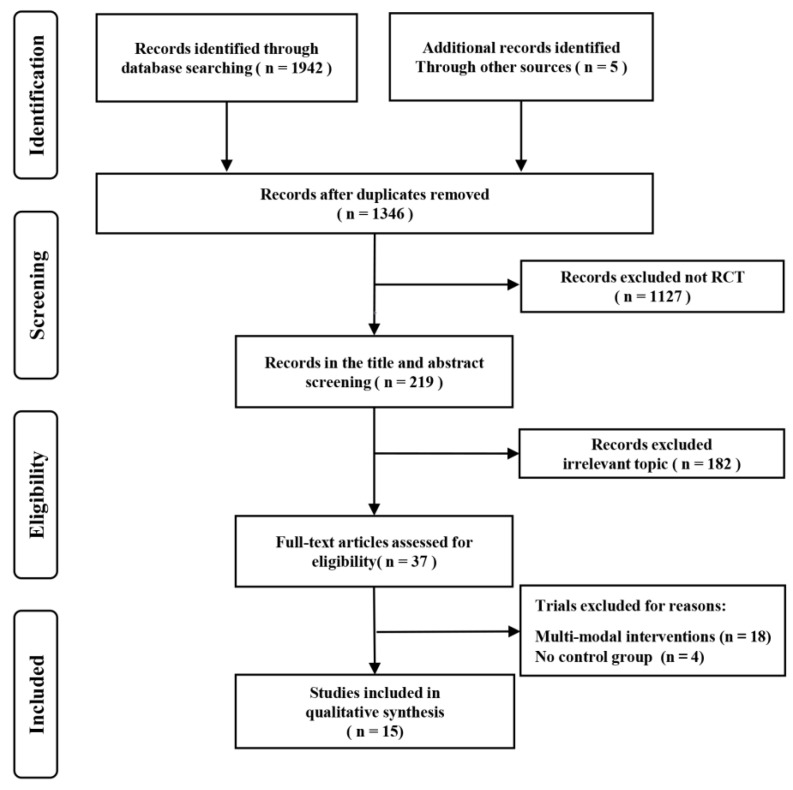
PRISMA flowchart of study selection.

As shown in Figure 1, a total of 1942 articles were initially retrieved from the databases and five records were retrieved from other sources [28,38,39,40,41]. A total of 1346 studies remained after excluding duplicates. After screening the titles and abstracts, 37 potentially eligible studies remained. Finally, 15 studies [20,27,28,38,39,40,41,42,43,44,45,46,47,48,49] examining the effect of aerobic exercise on cognitive function in AD patients were considered eligible for systematic review and meta-analysis after reading the full text.

### 3.2. Characteristics of the Included Studies

The main characteristics of the participants are shwon in Table S1. Of the fifteen studies, four were based in United States [45,47,48,49], two each in Denmark [43,46] and Italy [20,41], and one each in South Korea [44], China [27], Brazil [42], Canada [38], Germany [40], and France [28]. All included studies contained a sample size of 909 subjects diagnosed with AD. Aerobic exercise and control groups were composed of 503 and 406 subjects, respectively. The 15 included studies were published between 2002 and 2020. Of the 15 included studies, 4 studies performed moderate-intensity to high-intensity aerobic exercise [39,41,43,46], 3 studies did not describe exercise intensity [20,38,40], and the remaining included studies performed moderate-intensity aerobic exercise [27,28,42,44,45,47,48,49]. The overall duration varied from 9 weeks to 26 weeks, with an average intervention duration of 18.13 weeks. The frequency of interventions per week ranged from 2–5, with an average of 3.66 per week. The intervention time for each session varied from 30 to 60 min, with an average of 46.66 min per session. Interventions were administered at least 60 min per week, with an average weekly intervention time of 160 min.

### 3.3. Risk of Bias

**Figure 2:**
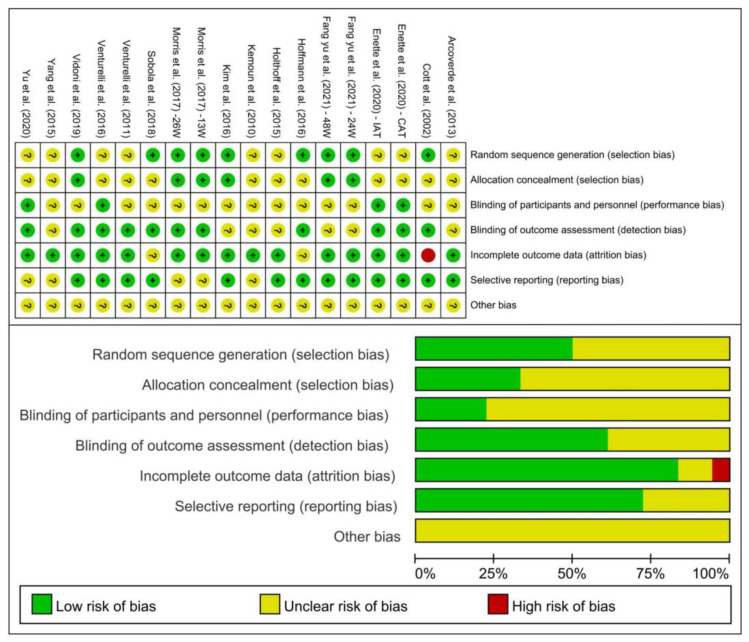
Results of Cochrane risk of bias tool [20,27,28,38,39,40,41,42,43,44,45,46,47,48,49].

**Figure 3:**
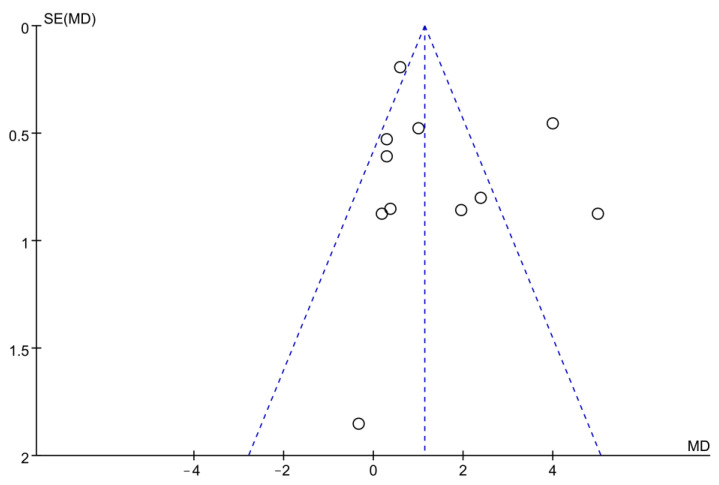
Funnel plot.

The quality of the included studies was evaluated using the Cochrane Collaboration tool according to the following six aspects: selection bias, performance bias, detection bias, attrition bias, reporting bias, and other bias. The quality of included studies is shown in Figure 2. Possible publication bias was assessed by examining the funnel plot (Figure 3), while the results of the Egger’s test indicated that the small sample size studies of the included study were not enough to affect the final results (*p* = 0.381, Table 1).

The standard assessment of the 15 studies included in this systematic review using the PEDro scale is shown in Table 2. Of the fifteen included studies, twelve were of good quality and three were of fair quality [34]. A limitation of the methodological quality of all studies was the lack of clarity on patients blinding. The mean PEDro score for all included studies was 6.33.

### 3.4. Meta-Analysis Results

#### 3.4.1. Effects of Aerobic Exercise on Cognitive Function in AD Patients

**Figure 4:**
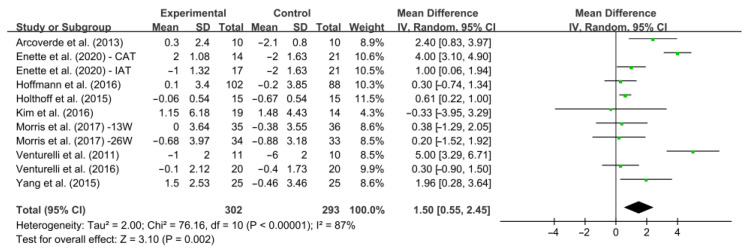
Meta-analysis results of the effect of aerobic exercise on cognitive function in AD patients [20,27,39,40,41,42,43,44,45].

After analyzing the data of all included studies, we found that aerobic exercise had a significant effect on increasing MMSE score in AD patients [WMD, 1.50 (95% CI, 0.55 to 2.45), *p* = 0.002, Figure 4], while there was significant heterogeneity (*I*^2^ = 87%). To explain the heterogeneity between eleven studies and find modifiable factors of aerobic exercise, four further subgroup analyses were further performed.

#### 3.4.2. Subgroup Analysis

**Figure 5:**
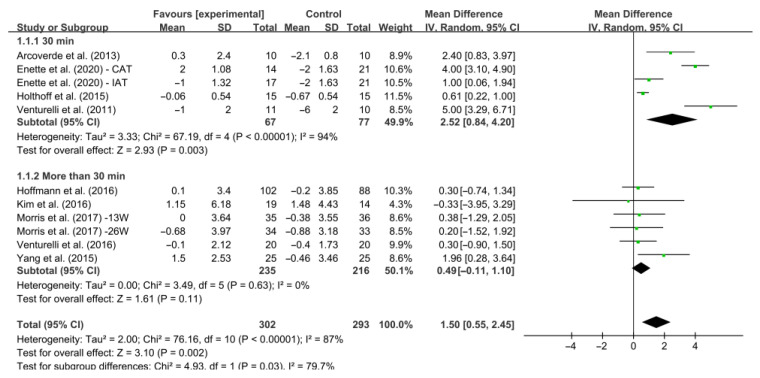
Meta-analysis results of the effect of the duration of intervention per session on cognitive function in AD patients [20,27,39,40,41,42,43,44,45].

Different results were observed when considering minutes of intervention per session (Figure 5). Specifically, interventions conducted 30 min per session increased MMSE score significantly [WMD, 2.52 (95% CI, 0.84 to 4.20), *I*^2^ = 94%, *p* = 0.003]. However, interventions conducted more than 30 min per session had no significant associations with MMSE score in AD patients [WMD, 0.49 (95% CI, −0.11 to 1.10), *I*^2^ = 0%, *p* = 0.11].

**Figure 6:**
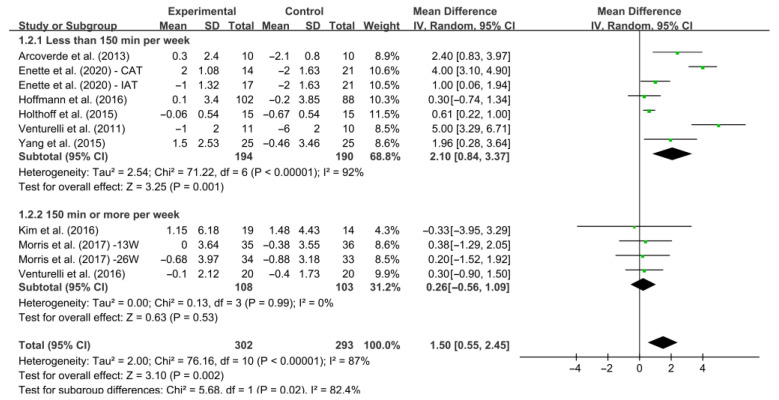
Meta-analysis results of the effect of the duration of intervention per week on cognitive function in AD patients [20,27,39,40,41,42,43,44,45].

In addition, different results were observed when considering minutes of intervention per week (Figure 6). Specifically, interventions conducted less than 150 min per week increased MMSE score significantly [WMD, 2.10 (95% CI, 0.84 to 3.37), *I*^2^ = 92%, *p* = 0.001]. However, interventions conducted 150 min or more per week exercise had no significant associations with MMSE score in AD patients [WMD, 0.26 (95% CI, −0.56 to 1.09), *I*^2^ = 0%, *p* = 0.53].

**Figure 7:**
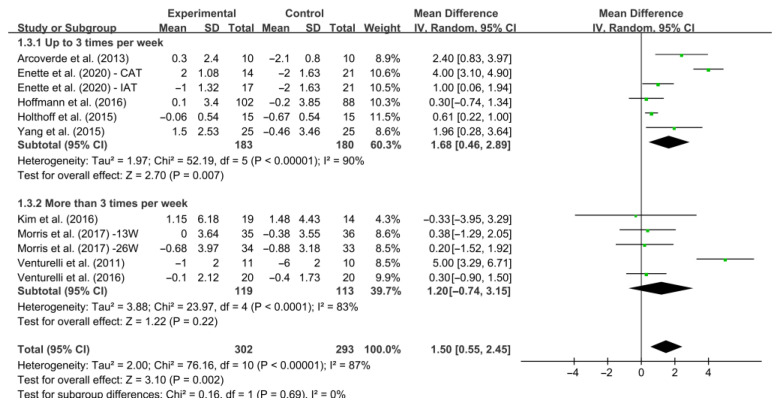
Meta-analysis results of the effect of the frequency of intervention per week on cognitive function in AD patients [20,27,39,40,41,42,43,44,45].

Furthermore, different results were observed when considering frequency of intervention per week (Figure 7). Specifically, interventions conducted up to three times per week increased MMSE score significantly [WMD, 1.68 (95% CI, 0.46 to 2.89), *I*^2^ = 90%, *p* = 0.007]. However, interventions conducted more than three times per week had no significant associations with MMSE scores in AD patients [WMD, 1.20 (95% CI, −0.74 to 3.15), *I*^2^ = 83%, *p* = 0.22].

**Figure 8:**
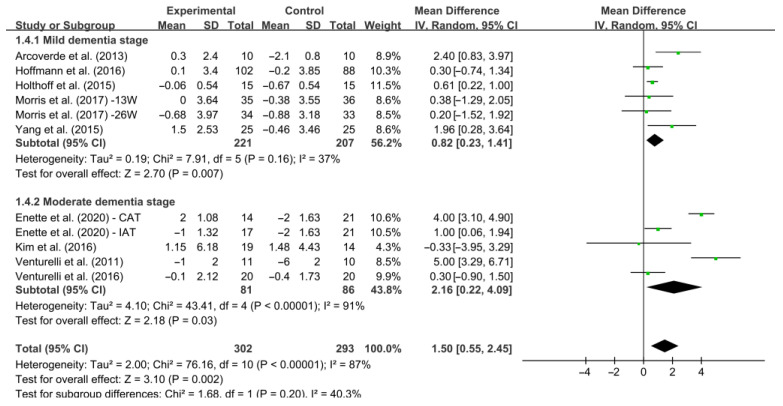
Meta-analysis results of the effect of aerobic exercise on cognitive function in mild or moderate AD patients [20,27,39,40,41,42,43,44,45].

Moreover, different results were observed when considering basal cognitive status of participants (Figure 8). Specifically, aerobic exercise increased MMSE score significantly in mild [WMD, 0.82 (95% CI, 0.23 to 1.41), *I*^2^ = 37%, *p* = 0.007] and moderate [WMD, 2.16 (95%CI, 0.22 to 4.09), *I*^2^ = 91%, *p* = 0.03] AD patients. Subgroup analysis indicated that a worse basal cognitive status was associated with greater improvement in MMSE score.

## 4. Discussion

The aim of this study was to explore the effect of aerobic exercise on cognitive function in AD patients. From 1942 search records initially identified, 15 studies were considered eligible for systematic review and meta-analysis. Our results showed that there was a significant effect of aerobic exercise on increasing MMSE score in AD patients. Subgroup analyses showed that interventions conducted 30 min per session, less than 150 min per week, and up to three times per week increased MMSE score significantly. In addition, a worse basal cognitive status was associated with greater improvement in MMSE score.

### 4.1. Effects of Aerobic Exercise on Cognitive Function

This systematic review and meta-analysis indicated that aerobic exercise had the potential to improve the cognitive function in AD patients, as manifested by an improvement in MMSE score. Our study showed that aerobic exercise contributed to an overall improvement in MMSE score by 1.50 (WMD), which was of clinical importance for AD patients. We noticed that under the theme of exercise intervention in AD patients, most exercise interventions were aimed at increasing the patients’ physical activity. The intervention methods included resistance, aerobic, balance, and combined exercise. Despite differences in exercise interventions, studies have demonstrated the link between aerobic exercise and improved cognitive function in AD patients [50,51,52].

Aerobic exercise can improve aerobic fitness, reduce the risk of chronic disease in older adults, and increase life expectancy. There are a growing number of studies suggesting that aerobic exercise can promote neuroplasticity, induce increased hippocampal neurogenesis [53,54], and protect against or reverse age-related hippocampal atrophy [55]. Regular exercise improves cerebrovascular and endothelial function, reduces oxidative stress and systemic inflammation, and helps regulate immune function, which may contribute to improved neuronal function [56,57,58]. Although the mechanism of aerobic exercise improving cognitive function had not been fully revealed, it was speculated that the beneficial effect of exercise on cognitive function might be strengthened through the following mechanisms.

Firstly, studies have shown that aerobic exercise can increase the level of brain-derived neurotrophic factor (BDNF), which is involved in neuroprotection and promotes cell survival, neurite outgrowth, and synaptic plasticity [59,60,61] associated with cognitive function. In addition, aerobic exercise can also increase the brain’ uptake of circulating insulin-like growth factor-1 (IGF-1), a factor that promotes neuronal differentiation of progenitor cells [60,62]. Secondly, studies have shown that the pathogenesis of AD may be related to circadian rhythm arrhythmia [63]. AD patients often exhibit sleep fragmentation and abnormal circadian rhythm, and aerobic exercise can help improve circadian rhythm and sleep quality in AD patients, which in turn improves cognitive function [57]. Finally, aerobic exercise can improve vagal regulation by increasing heart rate variability (HRV), the vagal control of the cardiovascular system is associated with activity in the prefrontal cortex, a brain structure that supports executive function [64,65], which is associated with cognitive function [66].

### 4.2. Subgroup Analysis

In the studies we included, aerobic exercise significantly increased MMSE score in AD patients, but the heterogeneity between groups was high. Therefore, we used subgroup analysis to interpret the results. In the subgroup analyses, we sought to determine the effects of minutes of intervention per week, minutes of intervention per session, frequency of intervention per week, and the basal cognitive status of participants.

Aerobic exercise has a dose-response relationship for health effects, and appropriate loading is the key to the health benefits of exercise [67]. The cognitive benefits of aerobic exercise appear to apply here as well. The American College of Sports Medicine (ACSM) and the American Heart Association (AHA) recommend that older adults engage in at least 30 min of moderate-intensity aerobic exercise 5 days a week or at least 20 min of vigorous-intensity aerobic exercise 3 days a week [68]. A recently published expert consensus recommends that the frequency of aerobic exercise for older adults is 20–60 min, 3–7 days per week [67]. However, our results showed that interventions conducted 30 min per session had a significant effect on improving cognitive function, while interventions conducted more than 30 min per session had no significant associations with MMSE score in AD patients, which was consistent with the results of Jia et al. [10], showing that interventions conducted up to 30 min per session showed greater effectiveness for improving cognition of patients compared to those conducted more than 30 min per session. Many studies have shown that 20 min per session can significantly improve cognitive function in older adults [17,69]. Exercise is too short to induce changes in the level of physical arousal, brain structure, and brain function, while a long duration may lead to excessive fatigue and will not induce brain plasticity [70]. Therefore, it is important to determine the duration for which such changes are most effective [37]. Future studies should elucidate the effect of intervention-based exercise duration.

The World Health Organization (WHO) recommends that all adults perform 150–300 min of moderate-intensity aerobic exercise, 75–150 min of vigorous-intensity aerobic exercise, or an equivalent combination of moderate-intensity and vigorous-intensity aerobic exercise per week [71]. The WHO indicated that this recommendation also applies to older adults. Our subgroup analysis showed that interventions conducted less than 150 min per week increased MMSE score significantly, while interventions conducted 150 min or more per week had no significant associations with MMSE score in AD patients, which was in consistent with the results of Groot et al. [72], showing that although both interventions conducted up to 150 min per week and more than 150 min per week yielded a positive effect on cognitive function, interventions conducted up to 150 min per week appeared to be more effective. One study showed that the minimum threshold for cognitive improvement in healthy older adults was 90 min of physical activity per week [73]. In addition, Jia et al. [10] reported that interventions conducted up to 120 min per week had a tendency to show greater effectiveness for improving cognition of AD patients compared to those conducted more than 120 min per week. Considering the physical activity capacity of older AD patients, most RCTs set the weekly interventions at up to 150 min to reduce participant dropout and potential risk [74], which may lead to a conservative trend in the number of studies with exercise interventions more than 150 min per week.

As for frequency of intervention per week, our results showed that interventions conducted up to three times per week increased MMSE score significantly, while interventions conducted more than three times per week had no significant associations with MMSE scores in AD patients, which was in consistent with the results of Jia et al. [10], showing that interventions conducted up to three times per week showed greater effect on improving cognition of AD patients compared to those conducted more than three times per week, which indicated that high-frequency interventions did not have better cognitive effects than low-frequency interventions [10]. In addition, Cai et al. [70] also showed that low-frequency exercise (one or two times per week) and moderate-frequency exercise (three or four times per week) had a positive exercise effect on working memory in older adults, while high-frequency exercise (≥ five times per week) had no such positive effect. Moreover, the effect size for older adults engaged in low-frequency exercise was larger than that for those engaged in moderate-frequency exercise or high-frequency exercise. We noted that the use of exercise intervention frequency alone cannot exclude the influence of other confounding factors, such as the duration of intervention per session and the duration of intervention per week, which may influence effects of aerobic exercise on cognitive function. Therefore, the best combination is yet to be discovered, and current evidence is insufficient to provide precise recommendations regarding the frequency of exercise interventions.

MMSE scores indicate the degree of cognitive impairment, including severe (0–10), moderate (10–20), mild (20–25), and questionably significant (25–30) [75]. In the above results, we suspected that the improvement of cognitive function by aerobic exercise was influenced by the basal cognitive status of participants, so this study divided the included studies into two subgroups, namely the mild group and moderate group. Our results showed that aerobic exercise increased MMSE score significantly in both mild and moderate AD patients, while a worse basal cognitive status was associated with greater improvement in MMSE score. The cognitive decline rate assessed by MMSE was 3 points per year, with the slowest rate of decline in mild patients, followed by moderate, and the fastest rate of decline in severe patients [76]. One previous study in patients with moderate cognitive impairment has reported a faster cognitive decline in the control group than in the intervention group [20], and thus the cognitive benefits of aerobic exercise can be determined more sensitively. However, since studies included in this systematic review and meta-analysis focused on mild and moderate AD patients, and studies focused on severe patients need to be cautious when referring to our findings.

### 4.3. Limitations of the Review

This study has some limitations that should be considered. Firstly, the included studies were all randomized controlled trials of aerobic exercise intervention, which could not be completely blinded. Therefore, in the process of quality evaluation, subjective factors would cause a certain degree of deviation. Secondly, some RCT research protocols did not describe the exercise intensity, so adequate statistics on the effect of aerobic exercise intensity on the cognitive function in AD patients were not available. Finally, the types of exercise interventions across the included studies were consistent, but the details of the interventions varied widely, which may be an important source of heterogeneity.

## 5. Conclusions

This systematic review and meta-analysis indicated that aerobic exercise, especially conducted 30 min per session, less than 150 min per week, and up to three times per week, contributed to improving cognitive function in AD patients. Additionally, a worse basal cognitive status contributed to more significant improvements in cognitive function.

## Figures and Tables

**Table 1:** Results of Egger’s test.

Std_EFF	Coef.	Std. Err.	t	*p* > |t|	95% CI
Slope	0.5688279	0.7304694	0.78	0.456	−1.083609, 2.221265
Bias	1.409892	1.53018	0.92	0.381	−2.051615, 4.871399

**Abbreviations:** Coef, coefficient; Std. Err, standard error; t, *t*-test statistic; *p*, probability; 95% CI, 95% Confidence Interval.

**Table 2:** Methodological assessment of randomized controlled trials included in the systematic review using the PEDro scale.

Study	A	B	C	D	E	F	G	H	I	J	K	Score
Arcoverde et al. (2013) [42]	Y	Y	Y	Y	N	N	N	Y	Y	Y	Y	7/10
Kim et al. (2016) [44]	Y	Y	Y	Y	N	N	N	Y	Y	Y	Y	7/10
Hoffmann et al. (2016) [43]	Y	Y	Y	Y	N	N	N	Y	Y	Y	Y	7/10
Yu et al. (2020) [48]	Y	Y	N	Y	N	N	Y	N	N	Y	Y	5/10
Yang et al. (2015) [27]	Y	Y	N	Y	N	N	N	Y	Y	Y	Y	6/10
Fang Yu et al. (2021) [49]	Y	Y	N	Y	N	N	Y	N	N	Y	Y	5/10
Sobol et al. (2018) [46]	Y	Y	N	Y	N	N	N	Y	Y	Y	Y	6/10
Vidoni et al. (2019) [47]	Y	Y	N	Y	N	N	Y	Y	Y	Y	Y	7/10
Morris et al. (2017) [45]	Y	Y	Y	Y	N	N	N	Y	Y	Y	Y	7/10
Cott et al. (2002) [38]	Y	Y	N	Y	N	N	Y	Y	Y	Y	Y	7/10
Holthoff et al. (2015) [40]	Y	Y	N	Y	N	N	N	Y	Y	Y	Y	6/10
Kemoun et al. (2010) [28]	Y	Y	N	Y	N	N	N	N	Y	Y	Y	5/10
Venturelli et al. (2011) [20]	Y	Y	Y	Y	N	N	N	Y	Y	Y	Y	7/10
Venturelli et al. (2016) [41]	Y	Y	N	Y	N	N	Y	Y	Y	Y	Y	7/10
Enette et al. (2020) [39]	Y	Y	N	Y	N	N	N	Y	Y	Y	Y	6/10

**Legend:** A, Eligibility criteria and source; B, Random allocation; C, Concealed allocation; D, Baseline comparability; E, Blinding of participants; F, Blinding of therapists; G, Blinding of assessors; H, Adequate follow-up (>85%); I, Intention-to-treat analysis; J, Between-group statistical comparisons; K, Reporting of point measures and measures of variability. Y, yes; N, no.

## Data Availability

Not applicable.

## Supplementary Materials

The following supporting information can be downloaded at: https://www.mdpi.com/article/10.3390/ijerph192315700/s1, Table S1: Baseline characteristics and intervention details of included studies. File S1: PRISMA 2020 checklist.
