# Supplementary material for: The Effect of Aerobic Exercise on Cognitive Function in People with Alzheimer’s Disease: A Systematic Review and Meta-Analysis of Randomized Controlled Trials
Source: Int J Environ Res Public Health. 2022 Nov 25;19(23):15700. doi: 10.3390/ijerph192315700 (PMC9736612; doi:10.3390/ijerph192315700)
Supplement: Supplementary file 1 [file ijerph-19-15700-s001.zip › Table S1.pdf]

Table S1. Baseline characteristics and intervention details of included studies

Study	Country	Sample size	Age (y), mean ± SD; median (range)	Diagnostic criteria	Cognitive impairment	Intervention	Setting	Trainer	Intervention Details for IG				Measurement Instrument
									Duration	Days per week	Minutes per session	Intensity	
Arcoverde et al. (2013)	Brazil	IG = 10<br>CG = 10	IG: 78.5 (64.0-81.2)<br>CG: 79.0 (74.7-82.2)	NINCDS-ADRDA criteria<br>NINDS-AIREN criteria	Mild	IG: treadmill walking<br>CG: usual treatment	Hospital	PT, PE	16 w	2	30	Moderate: 60% VO_{2max}	MMSE, CAMCOG, CDT, VF, Stroop
Kim et al. (2016)	South Korea	IG = 19<br>CG = 14	IG: 81.9 ± 7.0<br>CG: 80.9 ± 6.1	Clinical diagnosis of dementia	Moderat e	IG: lower-limb aerobic exercise using bike + MCP<br>CG: multi component intervention (MCP)	Nursing institution	PT, paramedic	24 w	5	60	Moderate: 40-60% HR_{max}	MMSE, ADAS-Cog, CDT
Hoffmann et al. (2016)	Denmark	IG = 107<br>CG = 93	IG: 69.8 ± 7.4<br>CG: 71.3 ± 7.3	NINCDS-ADRDA criteria	Mild	IG: aerobic exercise on ergometer bicycle, cross trainer, and treadmill<br>CG: usual treatment	Community	PT	16 w	3	60	Moderate-High: 70-80% HR_{max}	MMSE, SDMT, ADAS-Cog, VF, Stroop,
Yu et al. (2020)	USA	IG = 53<br>CG = 25	IG: 77.0 ± 6.6<br>CG: 78.9 ± 5.6	Clinical Dementia Rating scale	Moderat e	IG: aerobic exercise on a stationary recumbent bicycle<br>CG: stretching (attention control)	Elderly community	Trained staff	24 w	3	60	Moderate: 50-70% HRR or score of 9-15 on the Borg rating of perceived exercise (RPE) scale	ADAS-Cog
Yang et al. (2015)	China	IG = 25<br>CG = 25	IG: 72.00 ± 6.69<br>CG: 71.92 ± 7.28	NINDS-AIREN criteria	Mild	IG: cycling training<br>CG: health education	Rehabilitation Clinic	Professional therapist	12 w	3	40	Moderate: 70% HR_{max}	MMSE, ADAS-Cog,
Fang Yu et al. (2021)	USA	IG = 64<br>CG = 32	IG: 77.4 ± 6.6<br>CG: 77.5 ± 7.1	Clinical Dementia Rating scale	Mild	IG: cycling<br>CG: stretching	Gymnasium,<br>Elderly community	Trained staff	24 w	3	50	Moderate: 50-70% HRR or score of 9-15 on the Borg rating of perceived exercise (RPE) scale	ADAS-Cog
Sobol et al. (2018)	Denmark	IG = 26<br>CG = 29	IG: 69.2 ± 6.9<br>CG: 68.9 ± 7.2	NINCDS-ADRDA criteria	Mild	IG: aerobic exercise on ergometer bicycle, cross trainer, and treadmill<br>CG: usual care	Not mentioned	PT	16 w	3	60	Moderate-High: 70-80% HR_{max}	SDMT
Vidoni et al. (2019)	USA	IG = 33<br>CG = 32	IG: 74.1 ± 6.8<br>CG: 71.1 ± 8.8	Clinical Dementia Rating scale	Mild	IG: aerobic exercise<br>CG: ST	Professional sports organization	Certified Personal Coach	26 w	3-5	30-50	Moderate: 60-75% HRR	DAD
Morris et al. (2017)	USA	IG = 39<br>CG = 37	IG: 74.4 ± 6.7<br>CG: 71.4 ± 8.7	Clinical Dementia Rating scale	Mild	IG: aerobic exercise<br>CG: ST	Professional sports organization	Certified Personal Coach	26 w	3-5	30-50	Moderate: 60-75% HRR	DAD, MMSE

Study	Country	Sample size	Age (y), mean ± SD; median (range)	Diagnostic criteria	Cognitive impairment	Intervention	Setting	Trainer	Intervention Details for IG				Measurement Instrument
									Duration	Days per week	Minutes per session	Intensity	
Cott et al. (2002)	Canada	IG = 30<br>CG = 25	IG: 83.23 ± 8.34<br>CG: 79.78 ± 8.30	Medical diagnosis of AD	Severe	IG: walk and talk<br>CG: no intervention	Community	Trained staff	16 w	5	30	Not mentioned	FACSM
Holthoff et al. (2015)	Germany	IG = 15<br>CG = 15	IG: 72.40 ± 4.34<br>CG: 70.67 ± 5.41	NINCDS-ADRDA criteria	Mild	IG: lower body training on the movement trainer<br>CG: usual care	Community	Family caregiver	12 w	3	30	Not mentioned	MMSE
Kemoun et al. (2010)	France	IG = 20<br>CG = 18	IG: 82.0 ± 5.80<br>CG: 81.7 ± 5.10	DSM IV criteria	Moderate	IG: exercises on walking, equilibrium, and stamina.<br>CG: not practice any physical activities	Nursing home	Not mentioned	15 w	3	60	Moderate: 60-70% HRR	ERFC
Venturelli et al. (2011)	Italy	IG = 11<br>CG = 10	IG: 83.00 ± 6.00<br>CG: 75.00 ± 5.00	Clinical Dementia Rating scale	Moderate	IG: walking<br>CG: usual care	Hospital	Paramedic	24 w	4	30	Not mentioned	MMSE
Venturelli et al. (2016)	Italy	IG-AE = 20<br>CG = 20	IG-AE: 84.00 ± 7.00<br>CG: 84.00 ± 10.00	Clinical diagnosis of dementia	Moderate	IG: walking<br>CG: usual care	Hospital	Paramedic	12 w	5	60	Moderate-High: Not mentioned	MMSE
Enette et al. (2020)	France	IG-CAT=14<br>IG-IAT = 17<br>CG = 21	IG-CAT: 74 (68-83)<br>IG-IAT: 79 (75-82)<br>CG: 79 (75-84)	DSM IV criteria	Moderate	IG-CAT: continuous aerobic training<br>IG-IAT: interval aerobic training<br>CG: health education	Not mentioned	PT	9 w	3	30	Moderate: IG-CAT, 70% HRmax<br>Moderate-High: IG-IAT, 80% HRmax	MMSE

Note: Cognitive impairment level is classified by the average Mini-Mental State Exam score of participants (20 to 30: mild; 10 to 20: moderate; < 10: severe).

Abbreviations: IG, intervention group**;** CG, control group; w, week (s); y, years; SD, standard deviation; MCP, consisted of music therapy, art therapy, horticulture therapy, handicraft, recreational therapy, stretching, laughing therapy, and activity therapy; ST, stretching and toning (nonaerobic exercises that core strengthening, resistance bands, modified tai chi, and modified yoga); MMSE, Mini-Mental State Examination; CAMCOG, CAMCOG-CANDEX, Cambridge Examination for Mental Disorders of the Elderly; CDT: Clock Drawing Test; VF, verbal fluency test; Stroop, Stroop Color and Word test; ADAS-Cog, Alzheimer's Disease Assessment Scale-Cognitive Subscale; SDMT, Symbol Digit Modalities Test; DAD, Disability Assessment for Dementia; FACSM, Functional Assessment of Communication Skills Mental Subscale; ERFC, Rapid Evaluation of Cognitive Function; NINCDS-ADRDA, Neurological and Communicative Disorders and Stroke Alzheimer's Disease and Related Disorders Association; NINDS-AIREN, National Institute of Neurological Disorders and Stroke-Association Internationale pour la Recherche et l' Enseignement en Neurosciences; DSM IV, Diagnostic and Statistical Manual-fourth edition; PT, Physical therapist; PE, Physical educator
